# Study on the Effect of the Nucleophilicity of Amine Accelerators on the Process and Dielectric Properties of Epoxy Materials for Dry Bushing

**DOI:** 10.3390/polym17192655

**Published:** 2025-09-30

**Authors:** Huize Cui, Shuo Chen, Ruilu Guo, Chumeng Luo, Chong Zhang, Wenpeng Li, Yushun Zhao, Taisen Lu, Yanning Zhao

**Affiliations:** 1 China Electric Power Research Institute Corporation, Beijing 100192, China; jameschz@126.com (H.C.); grl18810663757@163.com (R.G.); chumengluo@gmail.com (C.L.); 18611602136@163.com (C.Z.); lwp1017@126.com (W.L.); 2 College of Electrical and Automation Engineering, Hefei University of Technology, Hefei 230009, China; yushunzhao@126.com (Y.Z.); lutaisen@126.com (T.L.); 18264780250@163.com (Y.Z.)

**Keywords:** amine accelerators, nucleophilicity, crosslinking density, gel time, dielectric properties

## Abstract

The impregnation and curing process of dry bushing requires the epoxy material for bushing to have a good process performance. In addition, the actual operating conditions of dry bushing put forward high requirements on the dielectric properties of the epoxy material. Amine accelerators can not only improve the technological properties of epoxy materials such as gel time and curing exothermic temperature rise by regulating the reaction rate of epoxy resin and anhydride curing agent, but also optimize the dielectric properties of epoxy materials by regulating the crosslinking density of epoxy materials. However, there are many types of amine accelerators, and the effects of amine accelerators with different nucleophilicity on epoxy materials vary greatly. In this paper, four kinds of amine accelerators with different nucleophilic ability were selected to study the influence of the nucleophilic ability of amine accelerators on the process and dielectric properties of epoxy materials. The results show that the stronger the nucleophilicity of the amine accelerator, the shorter the gel time of the epoxy mixture and the higher the exothermic temperature rise during curing, indicating a poorer processing performance. However, stronger nucleophilicity also endows the epoxy material with superior dielectric properties. Among them, the strong nucleophilic ability of TEA shortens the gel time of the material by 50% and increases the curing exothermic temperature rise by 55.3% compared with the weak nucleophilic ability of the DET epoxy system; the dielectric constant and dielectric loss of the material are reduced by 8.3% and 39.5%, respectively, and the breakdown strength is improved by 11.4%. This paper reveals the contradictory relationship between the process and dielectric performance of epoxy materials triggered by the difference in the nucleophilic ability of amine accelerators, and it also provides a new research idea for the improvement of the process and in the dielectric performance of epoxy materials for dry bushing.

## 1. Introduction

Dry bushing is a key component used for conductors to pass through walls or boxes and other isolation structures, playing the role of insulation and support; it is the “throat” of the DC transmission system and has an important impact on the safety and stability of the power grid [1,2,3,4]. The capacitor core is the core component of dry bushing, manufactured and processed from epoxy resin-impregnated insulation paper. The molding quality of the epoxy material directly determines the safe and stable operation of dry bushing [5,6,7]. However, the epoxy materials for dry bushing in China mainly rely on imports, and their localization has become a key technical challenge to be overcome in the field of electrical materials.

Epoxy materials for dry bushing need to fulfill both process performance and dielectric property requirements [8,9]. In terms of process performance, the capacitor core is made of epoxy resin-impregnated insulation paper, and the impregnating liquid is impregnated from the bottom up through the capillary effect; thus, the epoxy impregnating liquid needs to maintain a low-viscosity state for a long period of time, which puts high requirements on the process window of epoxy mixtures [10]. In addition, due to the large size of the capacitor core, it is easy to generate a large amount of heat during the curing and molding process, which leads to excessive internal curing residual stress, affecting the quality of the capacitor core. From the perspective of dielectric performance, the bushing is exposed to a coupled environment of high electric field, temperature changes, and mechanical stress for a long time during operation. Therefore, it is required that the material has a matching dielectric constant to achieve uniform distribution of the electric field and suppress partial discharge. At the same time, it is necessary to maintain low dielectric loss to maintain thermal stability and delay the thermal aging process. And high breakdown strength is the ultimate insulation capability reflected under the synergistic guarantee of the above performance.

Regarding the effect of amine accelerators on the various properties of epoxy materials, several studies exist. LI et al. [11] investigated the effect of DMP-30 on the curing rate and mechanical properties of epoxy resin and found that DMP-30 could effectively shorten the curing time of the epoxy resin, but the effect on the mechanical properties of epoxy materials had a duality, and the addition of 1% and 2% of DMP-30 improved the high-temperature performance and tensile strength, while the addition of 3% DMP-30 decreased the tensile strength. Wu Xiaojing et al. [12] synthesized waterborne resins using waterborne alkyd resins (ARs) and waterborne epoxy resins (ERs), and investigated the curing behavior of epoxy materials in the system of a BDMA accelerator by infrared spectroscopy and differential scanning calorimetry. The results showed that after holding the epoxy material at 130 °C for 30 min under the effect of a BDMA accelerator, the IR spectra showed that the absorption peak of the epoxy group at 914 cm^−1^ had disappeared, indicating that it had been fully cured. Cheng Xiulian et al. [13] investigated the effects of six accelerators, hexamethylenetetramine, triethylamine, triethanolamine, urea, N,N-dimethylformamide and triethylbenzylammonium chloride, on the curing rate of a dicyandiamide epoxy resin curing system. The results showed that the optimum dosage of the accelerators was 1.5~2.5%, and the storage time of the dicyandiamide epoxy resin curing system with the addition of triethylbenzylammonium chloride could reach more than 2 months at room temperature. From the above studies, it can be seen that amine accelerators can regulate the curing process performance of epoxy materials by reducing the activation energy of reaction, but at present there are still fewer studies on the dielectric properties of epoxy materials by accelerators at home and abroad.

This paper selected four amine accelerators with different nucleophilic abilities. Firstly, the size of their nucleophilicity was analyzed and verified through simulation. Furthermore, the influence of different accelerators’ nucleophilic abilities on the process and dielectric properties of epoxy materials was studied. The research work of this paper reveals the influence of the nucleophilic ability of amine accelerators on the various properties of epoxy materials, providing a reference for the selection of accelerators for epoxy insulation materials and the regulation of the process and dielectric properties of epoxy materials for dry bushing.

## 2. Materials and Methods

### 2.1. Materials

**Figure 1:**
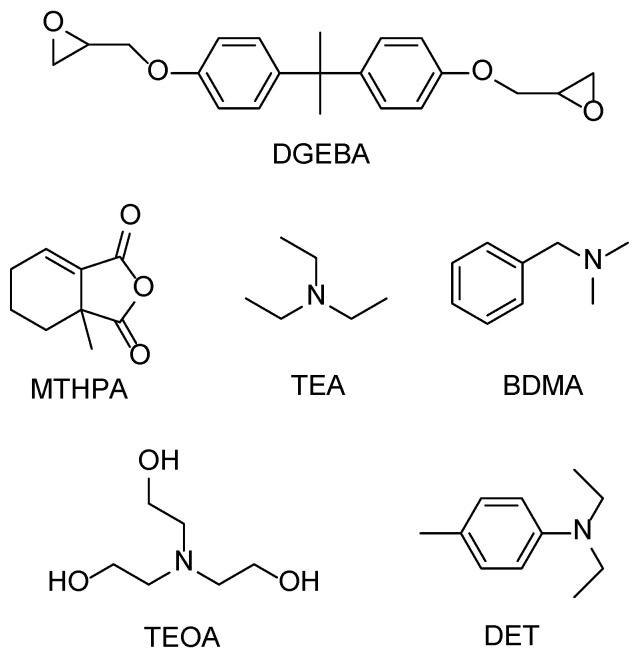
Chemical structure of main experimental raw materials.

The epoxy resin monomer molecule used is bisphenol A type epoxy resin (DGEBA, Sinopharm, Shanghai, China); the anhydride curing agent monomer molecule is methyl tetrahydrophthalic anhydride (MTHPA, Sinopharm, Shanghai, China); and four amine accelerators with different nucleophilic ability were used, respectively, triethylamine (TEA, Macklin, Shanghai, China), benzyl dimethyl amine (BDMA, Macklin, Shanghai, China), triethanolamine (TEOA, Macklin, Shanghai, China,), and diethyl-p-methyl aniline (DET, Macklin, Shanghai, China). The chemical structures of the main experimental raw materials are shown in Figure 1.

### 2.2. Methods

#### 2.2.1. Sample Preparation

**Figure 2:**
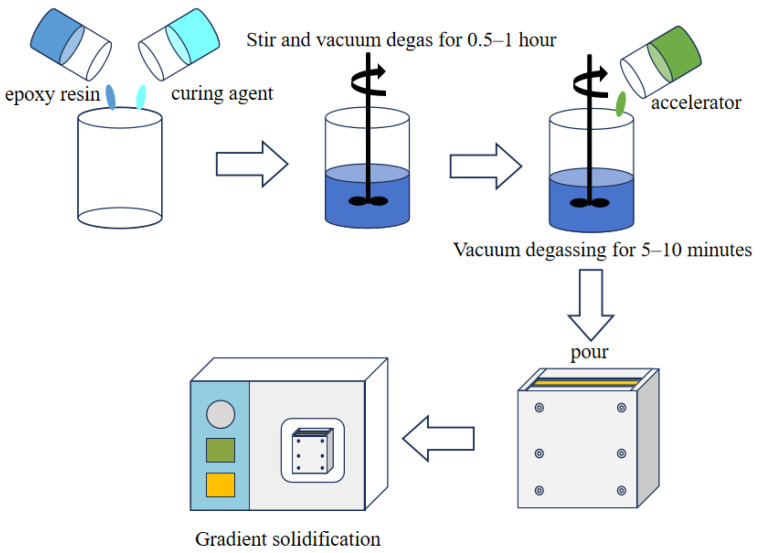
Schematic diagram for preparation of epoxy composite insulation material samples.

The experimental formulations for each group are shown in Table 1. In total, 100 g of epoxy resin and 88.91 g of methyl tetrahydrophthalic anhydride curing agent were weighed with an analytical balance in the reaction kettle, heated and stirred at 60 °C to homogeneously mix the two components, and degassed for 0.5~2 h. A total of 0.6 g of different kinds of amine accelerators was added to the reaction kettle and continued to be heated and degassed under the same conditions for 5~10 min. After degassing, the mixture was poured into a preheated mold at 80 °C and gradient curing was performed. The curing process was 80 °C/12 h + 120 °C/12 h. The schematic diagram of sample preparation is shown in Figure 2. After the curing process is completed, the mold is cooled to room temperature, and then the sample is demolded to obtain the epoxy composite insulating material sample. The performance of the sample is tested according to relevant testing standards.

#### 2.2.2. Gel Time

According to the international standard ISO 2555:2018 [14], a rotational viscometer was used to determine the viscosity–time characteristics of the epoxy mixture at 80 °C. The rotating torque was controlled at 10–90% of the maximum torque of the rotor, and the measurement was stopped when the epoxy mixture appeared to be drawn; the time required from the beginning to the end of the measurement is the gel time.

#### 2.2.3. Crosslinking Density

A TA Instruments DMA 800 Dynamic Thermomechanical Analyzer (TA instruments, New Castle, Delaware, USA) was used to test the crosslinking density of the epoxy material. Single cantilever mode was selected for the test, with a test frequency of 1 Hz, a maximum amplitude of 1 μm, a maximum dynamic force of 1 N, a temperature range of 40~200 °C, a temperature increase rate of 2 °C/min, and an air atmosphere. The loss angle tangent tanδ and the energy storage modulus versus temperature curves of the epoxy resin specimens were obtained, the peak temperature of the loss angle tangent was adopted as the Tg of the epoxy resin specimens, and the crosslinking density of the epoxy material was further calculated.

#### 2.2.4. Curing Exothermic Temperature Rise

A SIN–R7000C automatic temperature monitor (Sinomeasure, Hangzhou, China) was used as a temperature monitoring device for the epoxy material. The epoxy mixture was poured into a cylindrical container with a bottom diameter of 20 cm and a height of 30 cm inside the container, and a K-type thermocouple sensor end was placed at the geometric center of the epoxy resin to monitor the curing exothermic temperature rise at that point.

#### 2.2.5. Dielectric Constant and Dielectric Loss

According to the the international standard IEC 62631-2-1:2018 [15], we used Novocontrol concept 80 broadband dielectric spectroscopy (Novocontrol Technologies GmbH, Montabaur, Germany) to test the dielectric constant and dielectric loss of samples. The test specimen was a disc with a diameter of 100 mm and a thickness of 1 mm, the test frequency was 50 Hz, the temperature was 25 °C, and the voltage was 1 kV; each sample was tested 8 times and the test results were averaged.

#### 2.2.6. Breakdown Strength

According to the international standard IEC 60243-1:2013 [16], the test adopts the uniform voltage boosting method, and the pressurization rate is 100 V/s. In order to isolate the air and inhibit the occurrence of flashover discharge along the surface, the whole breakdown test device is submerged in insulating oil. The test temperature was 25 °C and the humidity was 50% RH.

#### 2.2.7. Molecular Dynamics Simulation

The molecular models of bisphenol A-type epoxy resin monomer, methyl tetrahydrophthalic anhydride curing agent monomer, bisphenol A-type epoxy resin/methyl tetrahydrophthalic anhydride crosslinking molecule monomer, and different amine accelerators were established and optimized geometrically in Materials Studio 2020.

In the DMol_3_ module, select the single-point energy calculation to compute the electron density and electrostatics of the molecular monomer. The hybrid functional is set as GGA, and the basis set is BLYP. After the calculation, use the Analysis tool in the DMol3 module to import the trajectory file, thereby obtaining the electrostatic potential distribution for various molecular models [17].

## 3. Results and Discussion

### 3.1. Influence of Nucleophilicity of Accelerators on the Crosslinking Reaction of Epoxy Materials

#### 3.1.1. Nucleophilicity of Amine Accelerators

**Figure 3:**
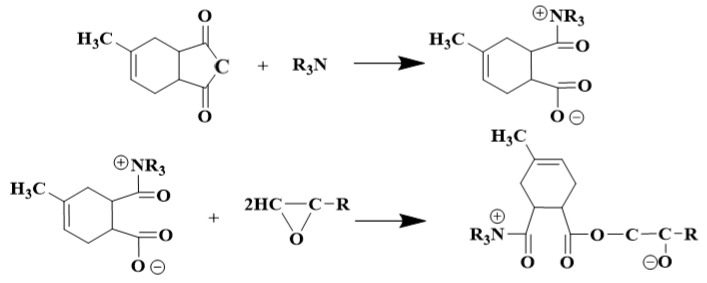
Crosslinking reaction principle of epoxy materials under amine accelerators.

Amine accelerators rely on the lone electron pair on the nitrogen atom so that they can attack the electron-deficient center and show nucleophilicity [18], which promotes the crosslinking reaction of epoxy materials as shown in Figure 3. First of all, amine accelerators promote the ring-opening reaction of the anhydride and generates active ion pairs. Subsequently, the epoxy group reacts with this ion pair to form an ester bond, and a new anion is generated. This new anion is able to further promote the ring-opening reaction of the anhydride, thereby initiating a chain reaction that ultimately leads to the formation of a polyester-type crosslinked structure [19]; the rate of crosslinking reaction of epoxy materials is altered by modulating the nucleophilic ability of the amine accelerators.

**Figure 4:**
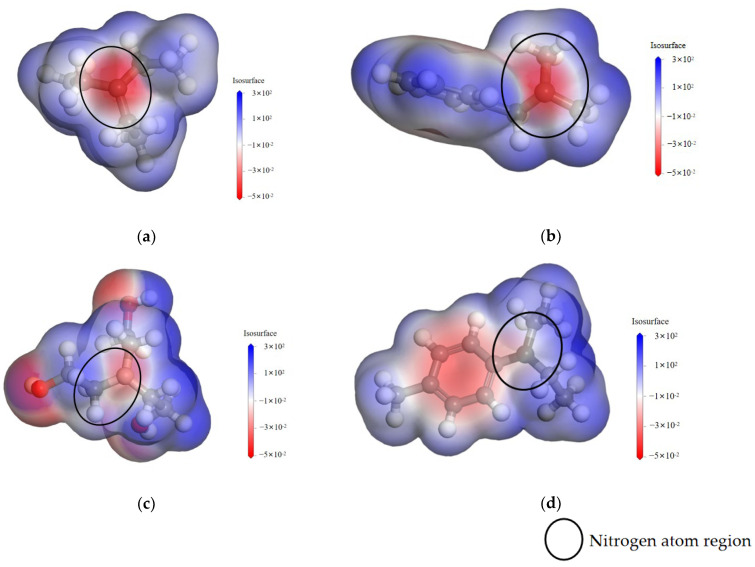
Electrostatic potential distribution of different amine accelerators. (**a**) Static potential distribution map of TEA. (**b**) Static potential distribution map of BDMA. (**c**) Static potential distribution map of TEOA. (**d**) Static potential distribution map of DET.

As can be seen from Figure 4, the nitrogen atom regions of different amine accelerators show different electrostatic potential distributions. Specifically, the color of the nitrogen atom region of TEA, BDMA, TEOA, and DET gradually changes from dark to light, and the corresponding electrostatic potential shows a trend from low to high. A lower electrostatic potential indicates a higher electron cloud density in the region, implying stronger nucleophilicity [20].

The nucleophilicity of nitrogen atoms on amine accelerators is mainly influenced by electronic and spatial site-resistance effects [21]. The electronic effect means that the more strongly the substituent group on the nitrogen atom gives electrons, the higher the density of the electron cloud on the nitrogen atom, and the easier it is to attack the anhydride molecule and make it ring-open. The spatial site-barrier effect means that the larger the space occupied by the substituent group on the nitrogen atom [22], the less accessible the anhydride molecule is to the nitrogen atom, which reduces the nucleophilicity of the amine accelerators. 

Under the influence of the spatial resistance effect, TEOA has three hydroxyethyl groups attached to the nitrogen atom, so its spatial resistance is the largest, followed by BDMA, and TEA has the smallest spatial resistance due to the fact that only three ethyl groups are attached to the nitrogen atom; thus, the nucleophilic abilities of the three accelerators are TEA > BDMA > TEOA. In addition, because DET is an aromatic amine and the amino group is directly connected to the benzene ring, by the electron conjugation effect, part of the electrons on the nitrogen atom are transferred to the benzene ring, which leads to a lower density of the electron cloud on the nitrogen atom, so its nucleophilicity is minimized.

The strength of the nucleophilic ability of amine accelerators as Lewis bases can be measured by the logarithmic value of the dissociation constant (Kb), pKb [23], and the pKb values of the four amine accelerators are shown in Table 2. The smaller the value of pKb, the greater the ability of the amine accelerators to give electrons in the catalytic reaction, and the greater the nucleophilic ability.

#### 3.1.2. Crosslinking Density

**Figure 5:**
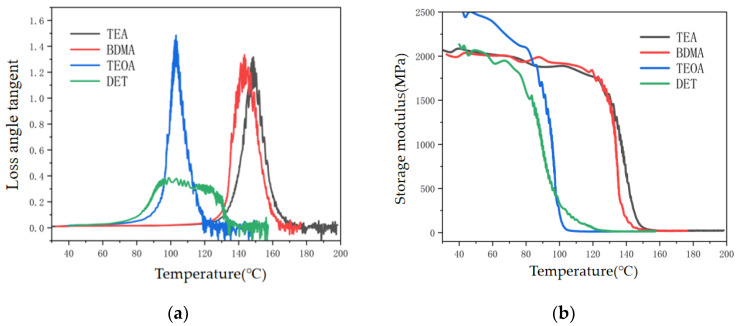
Loss tangent curve and storage modulus curve of epoxy insulation material. (**a**) Loss tangent temperature curve. (**b**) Storage modulus curve.

The crosslinking density of epoxy materials can reflect the number of crosslinking bonds per unit volume, which directly affects the physical and chemical properties of epoxy materials after the curing reaction [24]. As can be seen in Figure 5, the loss angle tangent and storage modulus of epoxy materials under different amine accelerators were tested by DMA to obtain the thermal performance parameters of different amine accelerator systems, and the test results are shown in Table 3. The crosslinking density of epoxy materials under different amine accelerators can be calculated according to Equation (1).

(1) $$\nu_{e}=\frac{E}{3R\;(T_{g}+40)}$$

where *V_e_* is the number of chains per unit volume, i.e., the network crosslinking density, with units of mol/m^3^; *T_g_* is the glass transition temperature; *E* is the storage modulus of the epoxy material corresponding to the temperature of (*T_g_* + 40) K in the DMA test; and *R* is the gas constant 8.314 J/mol·K [25].

**Figure 6:**
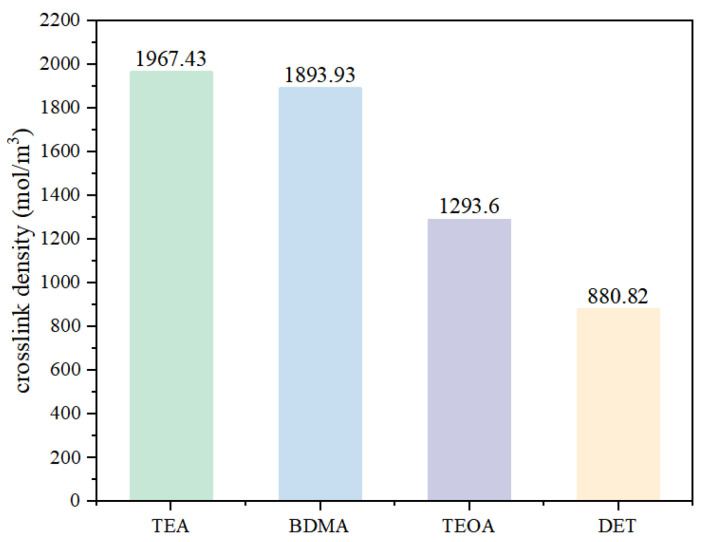
Crosslinking density of epoxy materials with different amine accelerators.

As can be seen from Figure 6, the stronger the nucleophilic ability of the amine accelerators, the higher the crosslinking density of the epoxy materials. The crosslinking density of the TEA system is 1967.43 mol/m^3^, while the crosslinking density of the DET system is only 880.82 mol/m^3^, which is 55.2% lower than that of the TEA system. It can be seen that the nucleophilic ability of amine accelerators not only affects the process of the crosslinking reaction of epoxy materials, but also has an effect on the crosslinking density of cured epoxy materials. This is due to the fact that as the curing reaction proceeds, the viscosity of the epoxy mixture continues to become greater, which restricts the chain segment movement of the epoxy molecules, resulting in the weak nucleophilic ability of the accelerator which makes it difficult for it to play a role, while the stronger nucleophilic ability of the accelerator is still able to promote the anhydride and the epoxy resin crosslinking reaction, and ultimately obtain an epoxy curing material with a higher crosslinking density.

### 3.2. Process Performance

#### 3.2.1. Gel Time Test Results

**Figure 7:**
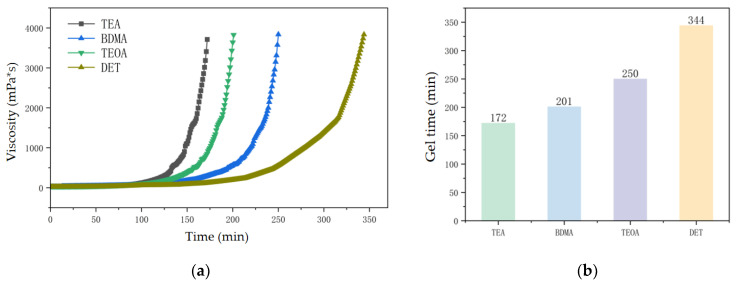
Process performance changes in epoxy mixtures with different amine accelerators. (**a**) Viscosity time characteristics of epoxy mixtures with different amine accelerators at 80 °C. (**b**) Gel time of Epoxy Mixtures with different amine accelerators at 80 °C.

Gel time is an important indicator of the working life and storage life of epoxy mixtures, which can be determined by measuring the change in viscosity of epoxy mixtures with time [26]. The viscosity–time characteristics of epoxy mixtures under different nucleophilic-capacity amine accelerator systems are shown in Figure 7a. The viscosity of epoxy mixtures under 80 °C increases with time, and when the viscosity of the mixtures increases to 3500–4000 mPa·s, the epoxy material appears to have a pulling phenomenon; the time corresponding to this point in time is the gel time of the mixtures [27]. This shows that the crosslinking reaction between epoxy resin and curing agent reaches the critical level of the gel point, and a three-dimensional infinite polymer network is formed in the system for the first time, leading to a sudden change in the macro properties of the material, from viscous liquid to non-flowing elastic gel. As can be seen from Figure 7b, the stronger the nucleophilic ability of the amine accelerators, the shorter the gel time of the epoxy mixture, in which the gel time of the DET system is up to 344 min, while the gel time of the TEA system is 172 min, which is 50% shorter than that of the DET system. This is due to the stronger nucleophilic ability of the accelerator which is more likely to promote the anhydride ring-opening reaction, thereby accelerating the crosslinking reaction rate and viscosity growth of the epoxy mixture, and ultimately shorten the gel time.

#### 3.2.2. Exothermic Temperature Rise for Curing

**Figure 8:**
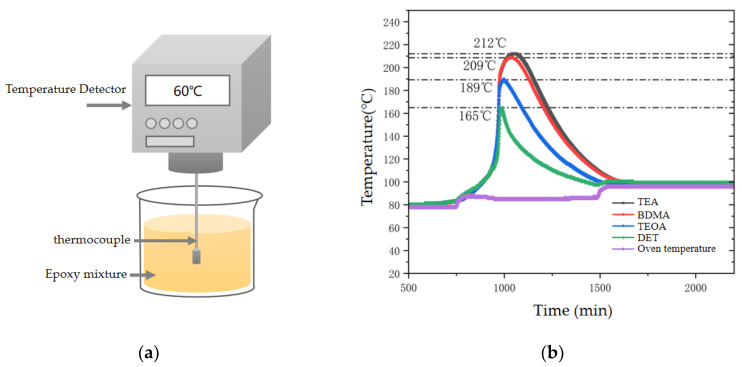
Curing heat release temperature rise of epoxy mixture. (**a**) Schematic diagram of temperature monitoring of epoxy mixture. (**b**) Curing heat release temperature rise of epoxy materials with different amine accelerators.

In order to characterize the curing exothermic temperature rise of epoxy materials with different nucleophilic abilities of amine accelerators, a temperature-monitoring platform was constructed in the laboratory to simulate the curing exothermic process of bushing capacitor cores. As shown in Figure 8a, the epoxy resin was poured into a cylindrical container, and the temperature changes during the curing process of the epoxy mixture were recorded in real time using a C7000 temperature monitor with a type K thermocouple. As can be seen from Figure 8b, the stronger the nucleophilicity of the amine accelerator, the higher the curing exothermic temperature rise of the epoxy material. When the oven setting temperature was 80 °C, the epoxy mixture started to be intensely exothermic, in which the highest exothermic temperature of the DET system was 165 °C, and the curing temperature rise was only 85 °C, whereas the highest exothermic temperature of TEA was as high as 212 °C, the curing temperature rise was 132 °C, and the curing exothermic temperature rise was increased by 55.3% compared with that of the DET system. The curing exothermic temperature rise of the epoxy mixture is mainly affected by its reaction rate: the stronger the nucleophilic ability of the accelerator, the faster the rate of crosslinking reaction of the epoxy material, and the more intense the exothermic effect. In addition, due to the large volume of the bushing capacitor core, when the exotherm is more intense, heat can easily accumulate inside the capacitor core, resulting in a further increase in the curing exothermic temperature rise.

### 3.3. Dielectric Properties

#### 3.3.1. Dielectric Loss and Dielectric Constant

**Figure 9:**
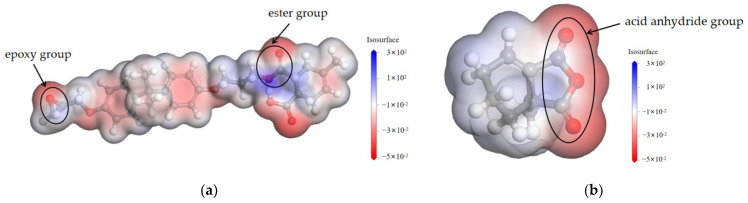
Distribution of electrostatic potential of different functional groups. (**a**) Distribution diagram of electrostatic potential between epoxy and ester groups. (**b**) Distribution diagram of electrostatic potential of anhydride groups.

As can be seen from Figure 9, there are differences in the distribution of electrostatic potential around different groups. The ester group region is the lightest in color and corresponds to the highest electrostatic potential, indicating that the electron cloud density in this region is lower and the nucleophilic ability is weaker [28]. In contrast, the regions of epoxy and anhydride groups are darker in color and have lower electrostatic potentials, indicating that these regions have higher electron cloud densities and stronger nucleophilic capabilities.

**Figure 10:**
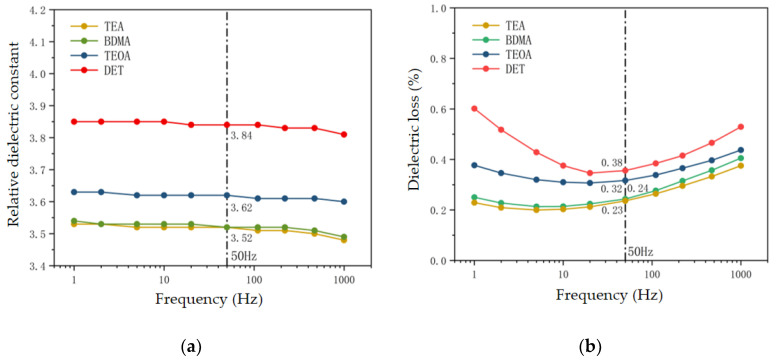
Dielectric constant and dielectric loss of epoxy materials with different amine accelerators. (**a**) Dielectric constant of epoxy materials with different amine accelerators. (**b**) Dielectric loss of epoxy materials with different amine accelerators.

As can be seen from Figure 10a, the stronger the nucleophilic ability of the amine accelerator, the smaller the dielectric constant of the epoxy material. At 50 Hz, the dielectric constant of the DET system is 3.84, while that of the TEA system is 3.52, which is a decrease of 8.3% compared with that of the DET system. Molecular dynamics simulations revealed that the ester groups generated by the crosslinking reaction of the epoxy materials were weak in polarity, while the unreacted epoxy and anhydride groups were strong in polarity. As the nucleophilic ability of the amine accelerator increases, the crosslinking density of the epoxy composites increases, leading to a decrease in the number of residual strong polar groups in the epoxy material, which results in a decrease in the dielectric constant of the material.

As can be seen from Figure 10b, the stronger the nucleophilic ability of the amine accelerator, the smaller the dielectric loss of the epoxy material in each frequency band. Taking 50 Hz as an example, the dielectric loss of the DET system is as high as 0.38%, while the dielectric loss of the TEA system is only 0.23%, which is 39.5% lower than that of DET. The dielectric loss of epoxy material mainly consists of two parts: conductivity loss and polarization loss [29]. Conductivity loss mainly occurs in the DC and low-frequency bands, which refers to the conductivity loss caused by the directional migration of epoxy material under the action of electric field [30]. Polarization loss mainly occurs in the high frequency range, referring to the loss caused by the flipping of dipoles in epoxy materials and friction and collision with molecular chains under the action of alternating electric fields [31]. When the nucleophilic ability of the accelerator is stronger, the crosslinking density of the epoxy material is higher, and the crosslinking network structure enhances the binding ability of the free electrons, which leads to the reduction in the conductivity loss of the epoxy material in the range of low frequency [32]. At the same time, with the increase in crosslinking density, the number of unreacted polar groups in the epoxy material is reduced, which leads to a reduction in the polarization loss of the epoxy material in the high-frequency band. It can be seen that the nucleophilic ability of amine accelerators not only affects the conductivity loss of the epoxy material, but also has a significant effect on its polarization loss, which ultimately leads to a reduction in the dielectric loss of the epoxy material in all frequency bands.

#### 3.3.2. Effect of Amine Accelerators on Breakdown Strength of Epoxy Materials

**Figure 11:**
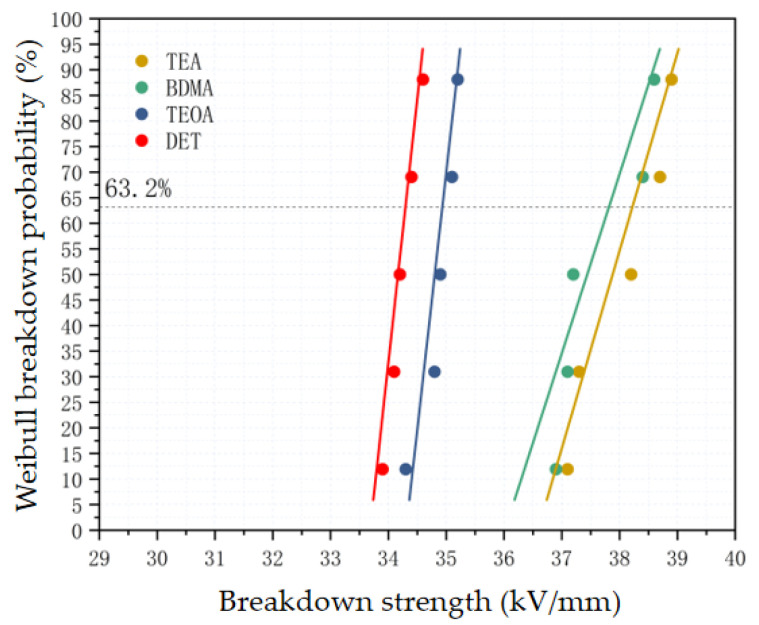
Breakdown strength of epoxy materials with different amine accelerators.

As can be seen from Figure 11, the stronger the nucleophilic ability of amine accelerators, the higher the breakdown strength of epoxy materials. Among them, the breakdown strength of the DET system is 34.3 kV/mm, while the breakdown strength of the TEA system is 38.2 kV/mm, which is 11.4% higher than that of the DET system. This is due to the fact that the stronger the nucleophilic ability of amine accelerators, the higher the crosslinking density of epoxy materials, and the smaller the percentage of free volume in the crosslinked network. A smaller free volume ratio can effectively hinder the movement of electrons within the material, thus reducing the average free range of electrons within the epoxy insulating material [33] and improving the breakdown strength of the epoxy material.

## 4. Conclusions

In this paper, four amine accelerators with different nucleophilic ability were used to investigate the effect of the nucleophilic ability of amine accelerators on the process and dielectric properties of epoxy materials through the characterization of epoxy materials under four systems, including crosslinking density, gel time, exothermic temperature rise of curing, dielectric loss, and breakdown strength.

(1) In terms of process properties, the stronger the nucleophilic ability of the amine accelerators, the shorter the gel time of the epoxy materials and the higher the exothermic temperature rise of curing. Specifically, the gel time of the TEA system was 50% shorter than that of the DET system, and the curing exothermic temperature rise was 55.3% higher. This is due to the fact that the stronger nucleophilic ability of the accelerator enables easier anhydride curing agent ring-opening, accelerating the epoxy resin and curing agent crosslinking reaction rate.
(2) From the dielectric properties, the stronger the nucleophilic ability of the amine accelerators, the lower the dielectric loss and dielectric constant of the epoxy material, and the higher the breakdown strength. The dielectric constant and dielectric loss of the TEA system were reduced by 8.3% and 39.5% compared with those of the DET system, and the breakdown strength was improved by 11.4%. This is attributed to the enhanced nucleophilicity of the accelerator which reduces the number of polar dipoles and at the same time restricts the movement of carriers in the crosslinked network, thus improving the dielectric properties of the epoxy material.
(3) The selection of amine accelerators with appropriate nucleophilic ability can, on the one hand, meet the process performance requirements in the manufacturing process of capacitor cores—that is, facilitate the molding operation and reduce the defects caused by insufficient impregnation. On the other hand, it can ensure that the cured capacitor core has excellent dielectric properties, thereby effectively enhancing the insulation strength of the dry bushing and making it more in line with the requirements of practical engineering applications.

## Figures and Tables

**Table 1:** Experimental formulas for each group.

Group	P-1	P-2	P-3	P-4
DRE331	100 g	100 g	100 g	100 g
MTHPA	88.91 g	88.91 g	88.91 g	88.91 g
TEA	0.6 g	0 g	0 g	0 g
BDMA	0 g	0.6 g	0 g	0 g
TEOA	0 g	0 g	0.6 g	0 g
DET	0 g	0 g	0 g	0.6 g

**Table 2:** The pKb values of different amine accelerators.

Group	TEA	BDMA	TEOA	DET
pKb	3.28	4.98	6.20	9.44

**Table 3:** Thermal performance parameters of different amine accelerator systems.

	TEA	BDMA	TEOA	DET
Tg(K)	420.55	415.35	385.85	381.95
Energy storage modulus E (MPa)	22.60	21.51	13.74	9.27

## Data Availability

The original contributions presented in this study are included in the article. Further inquiries can be directed to the corresponding authors.
